# Similarities and differences between service users’ and carers’ experiences of crisis resolution teams in Norway: a survey

**DOI:** 10.1186/s12888-022-03928-w

**Published:** 2022-04-14

**Authors:** Nina Hasselberg, Trude Gøril Klevan, Bente Weimand, Gunn-Marit Uverud, Katrine Høyer Holgersen, Johan Siqveland, Torleif Ruud

**Affiliations:** 1 Division of Mental Health Services, Akershus University Hospital, Lørenskog, Norway; 2 Faculty of Health and Social Sciences, University of South-Eastern Norway, Drammen, Norway; 3 University of South-Eastern Norway, Notodden, Vestfold Norway; 4 Nidelv Community Mental Health Center, Clinic of Mental Health, St Olavs hospital, Trondheim, Norway; 5 Department of Psychology, Norwegian University of Science and Technology, Trondheim, Norway; 6 National Center for Suicide Research and Prevention, University of Oslo, Oslo, Norway; 7 Institute of Clinical Medicine, University of Oslo, Oslo, Norway

**Keywords:** Crisis resolution teams, Acute, Emergency, Mental health crises, Service users, Carers, Experiences

## Abstract

**Background:**

Crisis resolution team (CRT) care in adult mental health services is intended to provide accessible and flexible short-term, intensive crisis intervention to service users experiencing a mental health crisis and involve their carers (next of kin). Research on users’ and especially carers’ experiences with CRT care is scarce and is mostly qualitative in nature.

**Methods:**

Altogether, 111 service users and 86 carers from 28 Norwegian CRTs were interviewed with The Service User and Carer Structured Interviews of the CORE Crisis Resolution Team Fidelity Scale Version 2. Their experiences with different aspects of CRT care were reported with descriptive statistics, and differences between service users’ and carers’ experiences were analyzed with the Mann-Whitney U Test.

**Results:**

The service users and carers reported that the CRT care mostly reflected their needs and what they wanted. The experiences of service users and carers were mostly similar, except for significant differences in received information and how the termination of CRT care appeared. Both groups experienced the organization of the CRT care as accessible, with continuity, reliability, and flexibility, but without a high intensity of care. Both groups found the content of the CRT care supportive, sensitive, with a choice of treatment type and a range of interventions beyond medication, but a lack of written treatment plans and discharge plans. Carers were rarely involved in discharge meetings. Regarding the role of CRTs within the care system, both groups agreed upon the lack of facilitation of early discharge from inpatient wards and lack of home treatment, but both groups confirmed some collaboration with other mental health services.

**Conclusion:**

Service users and carers found that the CRTs were accessible, reliable, flexible, supportive, sensitive, and provided a range of interventions beyond medication. Limitations were lack of a high intensity of care, limited written treatment and discharge plans, limited provision of home treatment, and lack of gatekeeping of acute beds. Both groups experienced the CRT care as mostly similar, but with significant differences regarding involvement in care planning and discharge preparation.

**Supplementary Information:**

The online version contains supplementary material available at 10.1186/s12888-022-03928-w.

## Introduction

During the past two decades, several Western countries have established crisis resolution teams (CRTs). They intend to offer accessible, short-term, intensive, 24/7 home treatment; provide a range of interventions; involve other mental health services and carers; and serve as an alternative to acute hospital admissions for service users experiencing an acute and severe mental health crisis [1, 2]. CRTs are multidisciplinary mental health teams, and team members with different specialist competences collaborate giving a complex intervention to multifaceted mental health crises [3, 4]. A full-scale CRT model has not been implemented in Norway, and Norwegian CRTs have shown diversity in organization, treatment philosophy, and practices  compared to UK [5–8].

With the development of crisis resolution teams to provide more community-based care for service users experiencing an acute mental health crisis, a crucial issue is how service users and carers experience CRT care. In a Cochrane review, evidence from one randomized control trial and other uncontrolled trials suggests that the introduction of CRT care is associated with increased satisfaction among both service users and carers compared to standard care [9–14]. However, in a cluster-randomized controlled trial with special training in the model for the intervention CRTs, service users’ satisfaction was not significantly higher compared to control CRTs without such training, even if the intervention CRTs achieved higher model fidelity and effects on services [15].

Despite inconclusiveness regarding critical aspects of the CRT model [13], service users have experienced several elements as important and helpful. Of particular importance were accessibility, safety, a holistic approach, relational aspects, the inclusion of carers, and collaboration with other mental health services; as service users valued help from CRTs for these reasons [14, 16, 17]. Although the need for practical support has also been documented [18, 19], such support has rarely occurred in a CRT context [3, 19].

With the explicit intention of providing contextual and multifaceted crisis intervention, CRTs aim to involve and collaborate with carers and other parts of informal and professional networks [1, 20–22]. However, according to Morant et al. [16], the original family and social systems approach to home-based crisis intervention appears to have been diluted in CRT practice. Several studies have found that many carers report a positive attitude toward community-based care, and this may be related to the accessibility of services and the opportunity for carers to be included during the provision of health services [23, 24]. Nonetheless, despite its arguably positive contributions, community-based care may increase the burden of caregiving if the received support and follow-up of carers are inadequate [25, 26]. This also appears to be the case in CRT care [16, 20]. Carers may express a lower level of satisfaction with crisis care than service users and wish for more intensive treatment of the service user and the possibility of being included by health personnel [24, 27, 28].

Research has shown that there are often contradictions between service users and carers regarding the service user’s mental health problems and what should be considered necessary treatment and follow-up. Confidentiality and lack of consent may contribute to a lack of communication and mutual understanding [29, 30]. A Norwegian evaluation of Assertive Community Treatment team leaders’ experiences with collaboration with relatives showed that the possibility of meeting the service users together with family members helped develop a mutual understanding for all parties [31]. Understanding home-based crisis treatment as a collaborative project involving professionals, service users, and carers, the similarities and differences of service user and carer experiences need to inform the further development of CRT practice. However, studies that include both service users’ and carers’ experiences perspectives are scarce.

Three domains salient for optimal care were identified in the large qualitative study of several stakeholders’ (service users, carers, practitioners, managers, and referrers) views about critical ingredients in CRT care [16]. First, the organization of CRT care should provide a rapid initial response, frequent home visits, be accessible, reliable, and flexible, and have continuity of staff. Second, the content of CRT care should include emotional support, involve the whole family, and offer a range of interventions. Third, the role of CRTs within the care system included being a gatekeeper for acute admissions.

### Aims

The aim of this study was to explore similarities and differences in how service users and carers experienced the care provided by Norwegian CRTs. The experiences have been explored related to three main areas of CRT care identified in the large qualitative study mentioned above [16]: the organization of the CRT, the content of the CRT care, and the role of the CRT within the system.

## Methods

### Study design

This quantitative explorative cross-sectional study was a part of the data collection for fidelity assessments of Norwegian CRTs in a multicenter study conducted in 2016 [6]. The study was done in collaboration with the Network of Acute Mental Health Services in Norway (akuttnettverket.no).

### Setting

The CRTs in Norway are a part of community mental health centers (CMHCs) with outpatient clinics, mobile teams, and local inpatient wards. Each of 19 health trusts includes mental health services with hospital departments and two to four CMHCs [31, 32]. The CRTs and other CMHC units also collaborate with general practitioners and primary health and social services run by the municipalities.

### Sample and recruitment

The sample of 111 service users and 86 carers (family members of service users) was recruited by the 28 CRTs participating in the multicenter study. These were half of the Norwegian CRTs identified in a previous survey [33]. The service users and carers were recruited independently, and it is unknown whether some of them were in the same family. The 28 CRTs from 15 health trusts responded to an invitation sent to all 19 health trusts in the country inviting their CRTs to participate in the study. The CRTs represented urban and rural areas in all four health regions.

### Measures

The service users were interviewed using Norwegian translations of The Service User Structured Interview, and the carers were interviewed using The Carer Structured Interview. These interviews were a part of the procedures to collect data on service users’ and carer’ experiences for the CORE Crisis Resolution Team Fidelity Scale Version 2 [2]. The two structured interviews in English are with approval from the developers of the fidelity scale included as supplementary files 1 and 2 to make the complete questions available for the readers. Both interviews had the same 69 questions, except four, but the wording was adapted for each informant group. They covered experiences of referrals, waiting time, accessibility, frequency and duration of meetings, information, treatment plans, reliability, inclusion of families and friends, medication, practical help, flexibility, respect, discharge, continuity, collaboration with other health services, and home visits. The structured interviews did not contain any questions on socio-demographics or other personal issues. Because the CRTs met the service users both in their homes and in the CRT location, we have used the term meeting in this study, and not consultation or visit. Most questions had a dichotomous response scale (yes/no), and some had three or more alternative responses. No data was recorded on the service users or carers participating in the interviews.

### Data collection and procedures

An evaluation team visited each CRT for 1 day to evaluate the team’s fidelity to the CRT model. The evaluation team consisted of three members: two clinicians and one service user researcher. The evaluators interviewed the team managers, team members, services users, carers, and managers from collaborating health services. Those data were only used in rating fidelity. The service user researcher conducted the interviews with service users and carers. These data were first included in rating fidelity, and then further analyzed in more detail in this study. Approximately half preferred to be interviewed by phone and half face to face during the evaluation team visit.

A month before the visit, the CRT staff was asked to recruit for interviews six service users and six carers seen by the CRT within the last 3 months. When approached by the staff, the service users and carers were informed about the purpose of the study and received written information about the study. The CRTs recruited, on average, four service users (range one to six) and four carers (range two to six). The staff found it somewhat harder to recruit carers than service users.

### Data analysis

Altogether, 46 of the 69 questions had a dichotomous response scale (yes/no). To obtain a unified structure for analyses, the rest of the questions and response scales were adjusted to dichotomous questions in this way: For 12 yes/no questions with “not relevant” as a third possible response, “not relevant” was recoded as missing. For nine non-dichotomous questions with three alternative responses, the two most similar responses were merged, and the question was rephrased to fit the dichotomous responses of yes/no. For two questions with more than three responses, the responses were merged into two groups, and the question was rephrased to fit the response of yes/no. Finally, each question was abbreviated and rephrased to fit both informant groups.

Differences between service users and carers in the percentage of “yes” responses were tested using the non-parametric Mann-Whitney U Test. To avoid type I errors due to the high number of questions to be tested, we set the significance level to *p* < .01. As the questions had dichotomous response scales, and with a varying number of missing values, it was not possible to do factor analyses to identify common dimensions for groups of questions and define possible subscales. However, we grouped the questions thematically based on their content. All analyses were done using SPSS for Windows version 27.

We organized and presented the results as one figure for each of the three main themes identified in the large qualitative study, which contributed to the content of the fidelity scale [16]. Each figure included diagrams showing a pair of bars for each question within the respective theme and organized in groups for subthemes. We also commented on and interpreted the results and organized the discussions around the same themes and subthemes. We chose to structure our study on the results with these three themes from the large qualitative study during development of the fidelity scale, rather than on the four fidelity subscales which in the development of the fidelity scale were identified based on concept mapping involving items and participants also from several other sources and stakeholder groups [2, 16].

## Results

### General satisfaction with the CRT care

Altogether, 93% of the service users and 89% of the carers responded yes to a question about their overall satisfaction with the CRT care (‘Did you or the one you care for find that the treatment and support reflected your needs and what you wanted?’).

### Experiences of organization of CRT care

**Fig. 1:**
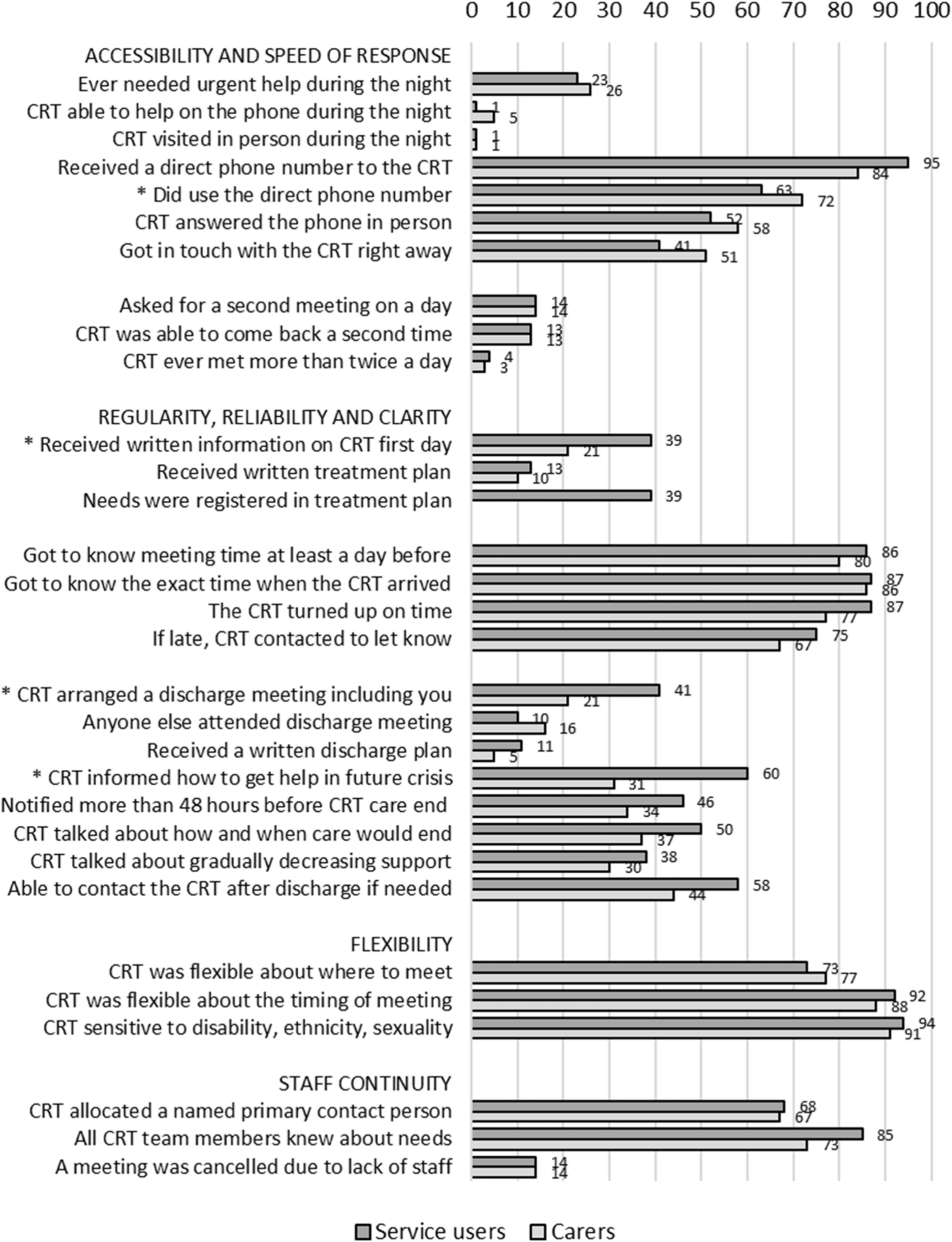
Service users’ (*N* = 111) and carers’ (*N* = 86) experiences of organization of crisis resolution teams’ care. Percentage of yes for each item. *) Items with significant differences (*p* < .01) between service users and carers

Figure 1 shows how the service users and carers experienced the organization of the CRT care. The CRTs were accessible during the operating hours and by phone, but not during the night, even if some service users and carers reported they had needed that. The CRT had been able to come back for a second time the same day for the few who had asked for this. The CRTs were predictable regarding time for meetings and flexible regarding where and when to meet.

The continuity of care was good, both regarding having a specific team member as a primary contact and in that the members in the team were updated on the service user’s situation and needs. For most service users, written information on the team and the treatment plan was not provided, and for more than half, the discharge preparation was limited.

### Experiences of content of CRTs care

**Fig. 2:**
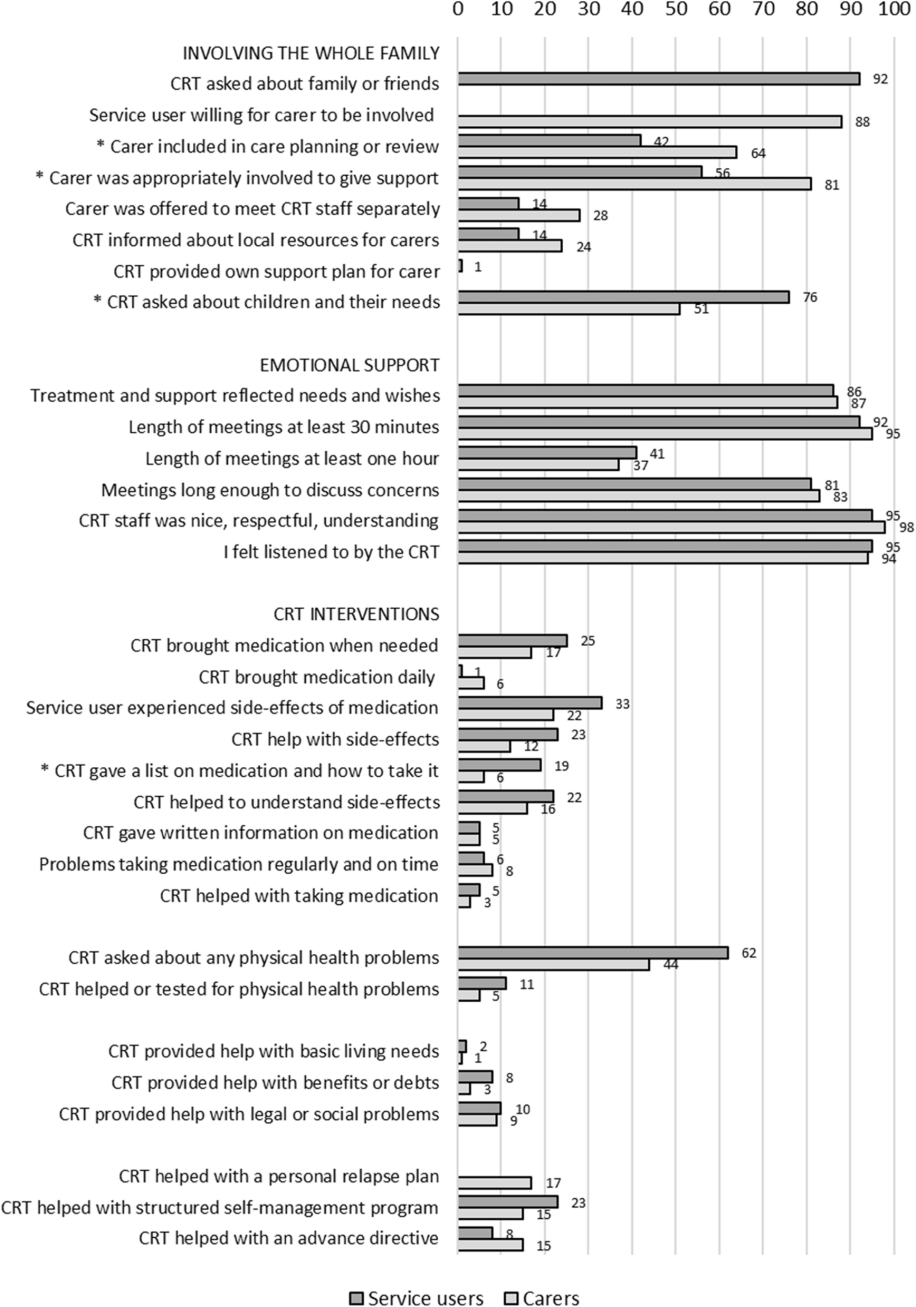
Service users’ (*N* = 111) and carers’ (*N* = 86) experiences of content of crisis resolution teams’ care. Percentage of yes for each item. *) Items with significant differences (*p* < .01) between service users and carers

Figure 2 shows how the service users and carers experienced the content of the CRT care. The CRTs had asked for information about family and friends, including children of the service user. Most service users were willing to have carers involved in the CRT care. Half or more of the carers were involved in care planning or review and giving the service user support. A few carers were offered the opportunity to meet the team separately or provided with information on local resources for carers, and one carer had been provided his/her own support plan.

The treatment and support mostly reflected needs and wishes, and the CRT staff was experienced as nice, respectful, and understanding. All service users and carers felt listened to, and the length of the meetings was experienced as long enough to discuss their concerns.

A minority of the services users were provided follow-up on medication or side effects of medication. More than half reported that the CRT asked about any physical health problems, and a few were helped or tested for physical health problems. Very few were provided help with basic needs, economic benefits or needs, or legal or social problems. Some were provided help regarding structured self-management or personal plans on what to do in case of future crisis or relapse.

### Experiences of the role of CRTs within the care system

**Fig. 3:**
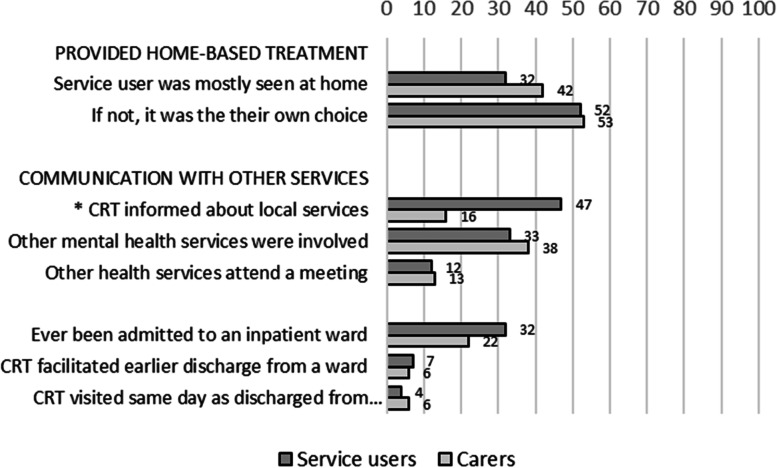
Service users’ (*N* = 111) and carers’ (*N* = 86) experiences of the role of crisis resolution team within the care system. Percentage of yes for each item. *) Items with significant differences (*p* < .01) between service users and carers

Figure 3 shows how the service users and carers experienced home-based treatment and CRTs’ collaboration with other services. Less than half of the services users were provided with home-based treatment, and if not, this was often their own choice. Half were informed about other local services. One-third reported that other health services were involved, but only a few experienced that other health services providers attended a meeting. The CRTs had little collaboration with inpatient units in the mental health services, such as facilitating early discharge for service users from inpatient wards.

### Similarities and differences in service users’ and carers’ experiences

As shown in the three figures, the service users’ and carers’ experiences of Norwegian CRT care were mostly similar. Significantly fewer carers than service users reported having received written information about the CRT on the first day, had been asked about children in the family and their needs, had received a list of prescribed medication, had attended a discharge meeting, or had been informed on access to crisis help in the future and other available local services or resources. However, significantly more carers than service users reported that they had used the CRT’s direct phone number and had been involved in care planning or reviews.

## Discussion

In summary, most of the service users and carers reported that the CRT care reflected their needs and what they wanted. They found the care to be accessible, reliable, flexible, supportive, sensitive to choices about treatment types not limited to medication, psychological interventions, and involvement of other mental health services to a certain extent. However, most service users and carers did not experience other core CRT elements, such as 24/7 availability, high emphasis of home treatment, high intensity of care, practical and social help, gatekeeping of acute inpatient beds, and facilitation of early discharge from inpatient wards. The service users’ and carers’ experiences of the CRT care were mostly similar, but with some significant differences, mainly regarding involvement in care planning, provision of information on the CRT and other services, and involvement in the discharge process.

### Experiences with organization of CRT care

High accessibility is a key element of CRT care. As reported in an earlier paper, we know that none of the CRTs in the study were operating 24 h, 7 days a week [6]. While 16 teams operated during extended hours on weekdays, and six of these also for some hours during weekends, the remaining 12 teams operated only during office hours on weekdays. Most needs during nights were expected to be met by GPs on call and other primary health services available 24/7. Still, an earlier study in Norway has shown that the likelihood of being admitted to an inpatient ward was significantly lower for patients treated by CRTs operating with extended hours than CRTs operating during office hours only [34].

High flexibility and reliability of CRTs regarding adjustment and keeping time for meetings were reported by most service users and carers. The same was reported for staff continuity, having a specific team member as a primary contact, and for team members being updated on the service user’s situation and needs.

Providing intensive care is also a key element of the CRT approach [1]. Our study confirmed previous findings of a lack of intensive care in Norwegian CRTs [6, 33]. We do not know if less intensive care was due to scarce resources or the treatment culture of the Norwegian CRTs, such as giving priority to longer meetings with the service users. Limited operating hours may also limit the provision of intensive care and CRT care for service users needing intensive care [6]. Service users and carers experienced the meetings as long enough to discuss their concerns, while sufficient time to talk was less provided in the UK [2]. Finding the balance between intensity of care and length of consultations might thus be important to optimize CRT care.

The lack of written treatment and discharge plans might reflect that CRT care is a short-term intervention and often a brief part of a longer care pathway involving other mental health services. However, the lack of written treatment and discharge plans in CRT care reflects a problem recognized in Norwegian mental health care, even though written treatment plans are statutory. CRTs may also find less need to write a treatment plan for service users who do not have a psychiatric disorder that needs further treatment after the CRT crisis intervention. Interviews with the Norwegian CRTs for the fidelity assessment showed that the teams did not use advance directives, which are not an established practice in Norway, but may collaborate with service users and carers forming written plans for actions in any future crises [6].

### Experiences with the content of CRT care

The fact that Norwegian CRTs are reported to provide long meetings with emotional support and not only a medication focus may be a result of the Norwegian CRTs being multidisciplinary, including psychiatrists, psychologists, nurses, and social workers [31]. Morant et al. highlighted the importance of keeping “the broad biopsychosocial focus of the original CRT model and not operate using a more biomedical approach, enacted in brief home visits focused on medication and risk management with less psychosocial input” [16].

However, psychological support to many and medication and practical help to few may also reflect the needs of the service users. Compared to the UK, the Norwegian CRTs have a smaller proportion of patients with severe mental illnesses [5]. This may lead to less need for practical support and medication management in Norwegian CRTs and give more time for talking, which also may be important to facilitate a therapeutic alliance. The types of practical support mentioned as examples in the structured interviews were basic living needs (getting food, heating, cleaning, repairs), help to achieve benefits or handle debts, and help with urgent legal or social problems (including employment, housing, etc.).

The low number of service users being offered help or tests for physical health problems may also reflect a lower proportion of service users with severe illnesses, but also that the service users continue to use their general practitioners for help with physical health care during the weeks they get help for a mental crisis from the CRT. On the other hand, it may also reflect a limited focus from the CRTs on the service users’ physical health [16].

### Experiences with the role of CRTs within the care system

Less than half of the service users reported being provided home-based CRT care, reflecting partly a substantial variation among Norwegian CRTs regarding providing home-based treatment [5, 6]. Contributing reasons for this may be level of resources (including limited operating hours), differences in treatment culture, differences in population density, and geographical distances between CRT location and service users’ homes [6]. However, service users with less severe mental problems may also have preferred meetings in the CRT location [6]. Some of the service users’ needs may also have been covered by other collaborating mental health services (like primary mental health care or other units at the community mental health center).

Some of the same factors, as well as not operating 24/7, may have contributed to the lack of gatekeeping of acute psychiatric beds and lack of facilitation of early discharge from inpatient wards [6]. However, the most important reason for this was probably that the national health authorities and the health trusts had not given the role of gate keeping acute beds to the crisis resolution teams [35].

### Comparison of service users’ and carers’ experiences

For most questions in our study, service users and carers reported similar experiences, and there were significant differences for only a few questions. Some of the ingredients agreed upon by both groups have been deemed important in CRT care, like a holistic approach, relational aspects, inclusion of other mental health services, and accessibility [14, 16, 17]. Most of the questions in the Service User and Carer Structured Interviews were about concrete aspects of CRT care, and the response options were mostly dichotomous, which may partly explain the high agreement between service users and carers as this gave less room for partial disagreements.

The current study showed differences in how service users and carers experienced the exchange of information and discharge from CRTs. While the service users more often took part in a discharge meeting and received information, the carers reported that they were seldom included in discharge meetings and received less information about local services and how to access crisis help in the future. This is in line with other studies exploring the role and experiences of carers [20, 28].

According to Lamb et al. [36], critical ingredients in CRT care, like crisis planning and support for carers, are rarely provided at optimal levels and are likely to lead to the CRT model not functioning as intended. Brennan et al. described that carers often experience being burdened with responsibility [28], while simultaneously, they are left out when important decisions are made that influence their lives and the person they care for [37]. Our finding that carers were rarely included in this part of the CRT care confirmed these earlier studies. This seems contradictory to findings that collaborative crisis services are important to improve outcomes for both carers and service users [28]. Furthermore, carers are considered an important part of home-based treatment. They often provide significant daily life support to persons experiencing mental distress and being at risk of a relapse of a mental health crisis [38, 39]. They may also evaluate the situation regarding whether assistance is required differently than do the service users [40]. Home-based services like CRT have carer involvement and contribution as a prerequisite, and it is surprising when carers are not included in the planning and preparation for discharge [28].

Our study showed that most of the service users and carers were given a direct phone number to the CRT and that more carers than service users had used this number. A direct phone number to reach the CRT is a key element of the CRTs’ accessibility when service users and carers need to speak to a mental health professional for support or advice. Our finding indicates that this key element is of particular importance to carers.

Service users received a list of medication significantly more often than did the carers. This may seem like a plausible finding, given the fact that medication is considered confidential. However, carers are sometimes involved in the follow-up of the service user’s daily medication, indicating that in some cases, the CRT staff may involve service users and carers in joint meetings on medication and side effects.

More carers reported that they were involved in care planning and support than service users reported that carers were. This may have been due to carers being recruited from a recent sample of CRT care episodes where carers were involved, while service users may have been recruited from the total sample of recent CRT episodes. On the other hand, the involvement reported by carers is compatible with the enthusiasm many Norwegian CRTs have shown for an open dialogue approach involving family and network [41].

Morant found that carers in the UK stood out in reporting that they felt excluded from care processes and that their views were often not considered [16]. It has proven to be important in a recovery process that service users and carers are actively involved in choice regarding types of treatment. Involvement and collaboration can also contribute to the transfer of experience-based knowledge from service users and carers to service providers.

### Strengths and limitations

One strength of the study was the larger number of service users and carers than in previous qualitative studies on service users’ and carers’ experiences with CRT care. Furthermore, the informants represented half of the Norwegian CRTs in most health trusts in both rural and urban areas in all health regions. This suggests that the data may be representative of service users and carers of such teams in Norway. However, the sample may have been biased toward positive experiences, as the service users and carers recruited by the CRT for interviews may have been among those with a predominantly good relationship to the CRT. Also, several raters participating in the evaluation team may have introduced some unknown variances in conducting the structured interviews, despite joint training and close collaboration between raters.

One strength is that two experts by experience were co-researchers and members of the research group. Being interviewed by an expert by experience may have made service users and carers feel safe and understood and made it easier for them to share their experiences. It may also have improved recruitment to interviews if the CRT told potential informants that experienced service users were among the researchers. The co-researchers also contributed to the interpretation and discussion of the results.

One limitation is the lack of information on sociodemographic or other characteristics of the service users and carers and the lack of information on who was related and had experienced the same CRT care episode. Differences in characteristics of CRT care episodes reported by service users and carers may also have influenced the comparison of their experiences in unknown ways. Lack of graded response scales may have limited the sensitivity to measure differences in experiences. The structured interviews with specific questions and closed response alternatives did not give the service users and carers any possibility to tell of other experiences or choose other alternative responses. The cross-sectional design of the study did not allow the research to draw any conclusion on causality. The study was conducted within a Norwegian context, which may limit generalizability to other contexts.

## Conclusion

Most of the service users and carers reported that the CRT care reflected their needs and what they wanted. The CRTs were accessible, reliable, flexible, supportive, sensitive, and with a range of interventions beyond medication. Limitations were lack of high intensity of care, limited written treatment and discharge plans, limited provision of home treatment, and lack of gatekeeping of acute beds. The service users’ and carers’ experiences of the CRT care were mostly similar, but with significant differences regarding involvement in care planning and discharge preparation.

## Supplementary Information

**Additional file 1.**

**Additional file 2.**

## Data Availability

The datasets used and/or analyzed during the current study are available from the corresponding author on reasonable request.

## Abbreviations

CRT: Crisis Resolution Team
CMHC: Community Mental Health Center
