# Supplementary material for: Similarities and differences between service users’ and carers’ experiences of crisis resolution teams in Norway: a survey
Source: BMC Psychiatry. 2022 Apr 14;22:266. doi: 10.1186/s12888-022-03928-w (PMC9011940; doi:10.1186/s12888-022-03928-w)
Supplement: Supplementary file 1 — Additional file 1. [file 12888_2022_3928_MOESM1_ESM.pdf]

CORE CRT Fidelity Review - Service User interview schedule

**Item 1**	**How long was it between being referred and seeing the Crisis Team to be assessed?**				
**SU1**	**SU2**	**SU3**	**SU4**	**SU5**	**SU6**
◯ Within 4 hours<br>◯ The same day<br>◯ Next day or later	◯ Within 4 hours<br>◯ The same day<br>◯ Next day or later	◯ Within 4 hours<br>◯ The same day<br>◯ Next day or later	◯ Within 4 hours<br>◯ The same day<br>◯ Next day or later	◯ Within 4 hours<br>◯ The same day<br>◯ Next day or later	◯ Within 4 hours<br>◯ The same day<br>◯ Next day or later

**Item 3**	**Who referred you to the Crisis Team?**				
**SU1**	**SU2**	**SU3**	**SU4**	**SU5**	**SU6**
◯ Staff on a Ward you were on<br>◯ Yourself<br>◯ Family or friends<br>◯ A&E<br>◯ GP<br>◯ Another service:	◯ Staff on a Ward you were on<br>◯ Yourself<br>◯ Family or friends<br>◯ A&E<br>◯ GP<br>◯ Another service:	◯ Staff on a Ward you were on<br>◯ Yourself<br>◯ Family or friends<br>◯ A&E<br>◯ GP<br>◯ Another service:	◯ Staff on a Ward you were on<br>◯ Yourself<br>◯ Family or friends<br>◯ A&E<br>◯ GP<br>◯ Another service:	◯ Staff on a Ward you were on<br>◯ Yourself<br>◯ Family or friends<br>◯ A&E<br>◯ GP<br>◯ Another service:	◯ Staff on a Ward you were on<br>◯ Yourself<br>◯ Family or friends<br>◯ A&E<br>◯ GP<br>◯ Another service:

Item 5	i) Did you ever need urgent help during the night from the Crisis Team?<br>ii) If Yes – were they able to help you on the phone?<br>iii) Did they ever offer to visit in person during the night?					
SU1		SU2	SU3	SU4	SU5	SU6
i) ◯ Yes ◯ No<br>ii) ◯ Yes ◯ No<br>iii) ◯ Yes ◯ No		i) ◯ Yes ◯ No<br>ii) ◯ Yes ◯ No<br>iii) ◯ Yes ◯ No	i) ◯ Yes ◯ No<br>ii) ◯ Yes ◯ No<br>iii) ◯ Yes ◯ No	i) ◯ Yes ◯ No<br>ii) ◯ Yes ◯ No<br>iii) ◯ Yes ◯ No	i) ◯ Yes ◯ No<br>ii) ◯ Yes ◯ No<br>iii) ◯ Yes ◯ No	i) ◯ Yes ◯ No<br>ii) ◯ Yes ◯ No<br>iii) ◯ Yes ◯ No

**Item 2b**	**i) Were you given a direct phone number to contact the Crisis Team?**
**Item 9a, 9c, 9d**	**ii) Did you use it?**<br>**iii) If Yes - did the Crisis Team answer the phone in person?**<br>**iv) If Yes – how quickly were you able to talk to clinical staff?**

SU1	SU2	SU3	SU4	SU5	SU6
i) ◯ Yes ◯ No	i) ◯ Yes ◯ No	i) ◯ Yes ◯ No	i) ◯ Yes ◯ No	i) ◯ Yes ◯ No	i) ◯ Yes ◯ No
ii) ◯ Yes ◯ No	ii) ◯ Yes ◯ No	ii) ◯ Yes ◯ No	ii) ◯ Yes ◯ No	ii) ◯ Yes ◯ No	ii) ◯ Yes ◯ No
iii) ◯ Yes ◯ No	iii) ◯ Yes ◯ No	iii) ◯ Yes ◯ No	iii) ◯ Yes ◯ No	iii) ◯ Yes ◯ No	iii) ◯ Yes ◯ No
iv) ◯ Straightaway<br>◯ within 20 minutes<br>◯ within 1 hour<br>◯ more than 1 hour	iv) ◯ Straightaway<br>◯ within 20 minutes<br>◯ within 1 hour<br>◯ more than 1 hour	iv) ◯ Straightaway<br>◯ within 20 minutes<br>◯ within 1 hour<br>◯ more than 1 hour	iv) ◯ Straightaway<br>◯ within 20 minutes<br>◯ within 1 hour<br>◯ more than 1 hour	iv) ◯ Straightaway<br>◯ within 20 minutes<br>◯ within 1 hour<br>◯ more than 1 hour	iv) ◯ Straightaway<br>◯ within 20 minutes<br>◯ within 1 hour<br>◯ more than 1 hour

**Item 9e**	**i) Did you ever ask the Crisis Team to come back a second time on a day when they had already visited you?**<br>**ii) If Yes – were they able to do this?**

SU1	SU2	SU3	SU4	SU5	SU6
i) ◯ Yes ◯ No	i) ◯ Yes ◯ No	i) ◯ Yes ◯ No	i) ◯ Yes ◯ No	i) ◯ Yes ◯ No	i) ◯ Yes ◯ No
ii) ◯ Yes ◯ No	ii) ◯ Yes ◯ No	ii) ◯ Yes ◯ No	ii) ◯ Yes ◯ No	ii) ◯ Yes ◯ No	ii) ◯ Yes ◯ No

**Item 12a**	**i) When the Crisis Team saw you initially did they give you information about their role and how they would help you?**
**Item 12a**	**ii) Was any of this information in writing?**
**Item 12b**	**iii) Did they give you a written treatment plan?**
**Items 12c, 12d**	**iv) Generally, did they let you know in advance when they would be visiting you?**
**Item 12e**	**v) Did they turn up reasonably on time (within an hour)?**
**Item 12f**	**vi) If they were going to be late, did they contact you to let you know?**

	**SU1**	**SU2**	**SU3**	**SU4**	**SU5**	**SU6**
i)	◯ Yes ◯ No	◯ Yes ◯ No	◯ Yes ◯ No	◯ Yes ◯ No	◯ Yes ◯ No	◯ Yes ◯ No
ii)	◯ Yes ◯ No	◯ Yes ◯ No	◯ Yes ◯ No	◯ Yes ◯ No	◯ Yes ◯ No	◯ Yes ◯ No
iii)	◯ Yes, within 4 days<br>◯ Yes, but later<br>◯ No	◯ Yes, within 4 days<br>◯ Yes, but later<br>◯ No	◯ Yes, within 4 days<br>◯ Yes, but later<br>◯ No	◯ Yes, within 4 days<br>◯ Yes, but later<br>◯ No	◯ Yes, within 4 days<br>◯ Yes, but later<br>◯ No	◯ Yes, within 4 days<br>◯ Yes, but later<br>◯ No
iv)	◯ At least a day before<br>◯ On the same day<br>◯ Turned up by surprise	◯ At least a day before<br>◯ On the same day<br>◯ Turned up by surprise	◯ At least a day before<br>◯ On the same day<br>◯ Turned up by surprise	◯ At least a day before<br>◯ On the same day<br>◯ Turned up by surprise	◯ At least a day before<br>◯ On the same day<br>◯ Turned up by surprise	◯ At least a day before<br>◯ On the same day<br>◯ Turned up by surprise
v)	◯ Yes ◯ No	◯ Yes ◯ No	◯ Yes ◯ No	◯ Yes ◯ No	◯ Yes ◯ No	◯ Yes ◯ No
vi)	◯ Always<br>◯ Usually<br>◯ Never	◯ Always<br>◯ Usually<br>◯ Never	◯ Always<br>◯ Usually<br>◯ Never	◯ Always<br>◯ Usually<br>◯ Never	◯ Always<br>◯ Usually<br>◯ Never	◯ Always<br>◯ Usually<br>◯ Never

**Item 13**	**i) Did the Crisis Team try to find out if you had any family or friends who could give you some support?**
**Item 13c**	**ii) Did any of these come to a care planning or review meeting with you and the Crisis Team?**
**Item 13e**	**iii) Did you feel that those identified were appropriately involved?**

**SU1**	**SU2**	**SU3**	**SU4**	**SU5**	**SU6**
i) ◯ Yes ◯ No<br>ii) ◯ Yes ◯ No<br>iii) ◯ Yes ◯ No	i) ◯ Yes ◯ No<br>ii) ◯ Yes ◯ No<br>iii) ◯ Yes ◯ No	i) ◯ Yes ◯ No<br>ii) ◯ Yes ◯ No<br>iii) ◯ Yes ◯ No	i) ◯ Yes ◯ No<br>ii) ◯ Yes ◯ No<br>iii) ◯ Yes ◯ No	i) ◯ Yes ◯ No<br>ii) ◯ Yes ◯ No<br>iii) ◯ Yes ◯ No	i) ◯ Yes ◯ No<br>ii) ◯ Yes ◯ No<br>iii) ◯ Yes ◯ No

<table>
<tr><td>Item 15b, 15c</td><td colspan="6">i) Did the Crisis Team bring you medication when you needed it?</td></tr>
<tr><td>Item 15c</td><td colspan="6">ii) How often did they bring you medication?</td></tr>
<tr><td colspan="2">SU1</td><td>SU2</td><td>SU3</td><td>SU4</td><td>SU5</td><td>SU6</td></tr>
<tr><td colspan="2">i) ◯ Yes ◯ No
◯ Not applicable

ii) ◯ Daily
◯ More than once daily
◯ Occasionally

iii) ◯ Yes ◯ No</td><td>i) ◯ Yes ◯ No
◯ Not applicable

ii) ◯ Daily
◯ More than once daily
◯ Occasionally

iii) ◯ Yes ◯ No</td><td>i) ◯ Yes ◯ No
◯ Not applicable

ii) ◯ Daily
◯ More than once daily
◯ Occasionally

iii) ◯ Yes ◯ No</td><td>i) ◯ Yes ◯ No
◯ Not applicable

ii) ◯ Daily
◯ More than once daily
◯ Occasionally

iii) ◯ Yes ◯ No</td><td>i) ◯ Yes ◯ No
◯ Not applicable

ii) ◯ Daily
◯ More than once daily
◯ Occasionally

iii) ◯ Yes ◯ No</td><td>i) ◯ Yes ◯ No
◯ Not applicable

ii) ◯ Daily
◯ More than once daily
◯ Occasionally

iii) ◯ Yes ◯ No</td></tr>
</table>

**Item 16d**	**i) Did they give you a list of what medication you should be taking and when?**
**Item 16e**	**ii) Did the Crisis Team help you to understand any medication you were prescribed – such as what it was for or any possible side-effects?**
**Item 16e**	**iii) Did they give you any of this information in writing?**
**Item 16f**	**iv) Did you have any problems with taking your medication regularly and on time?**
**Item 16f**	**v) If Yes - did the Crisis Team do anything to help you with this?**

**SU1**	**SU2**	**SU3**	**SU4**	**SU5**	**SU6**
i) ◯ Yes ◯ No<br>◯ Not applicable<br><br>ii) ◯ Yes ◯ No<br>◯ Not applicable<br><br>iii) ◯ Yes ◯ No<br>◯ Not applicable<br><br>iv) ◯ Yes ◯ No<br>◯ Not applicable<br><br>v) ◯ Yes ◯ No<br>◯ Not applicable	i) ◯ Yes ◯ No<br>◯ Not applicable<br><br>ii) ◯ Yes ◯ No<br>◯ Not applicable<br><br>iii) ◯ Yes ◯ No<br>◯ Not applicable<br><br>iv) ◯ Yes ◯ No<br>◯ Not applicable<br><br>v) ◯ Yes ◯ No<br>◯ Not applicable	i) ◯ Yes ◯ No<br>◯ Not applicable<br><br>ii) ◯ Yes ◯ No<br>◯ Not applicable<br><br>iii) ◯ Yes ◯ No<br>◯ Not applicable<br><br>iv) ◯ Yes ◯ No<br>◯ Not applicable<br><br>v) ◯ Yes ◯ No<br>◯ Not applicable	i) ◯ Yes ◯ No<br>◯ Not applicable<br><br>ii) ◯ Yes ◯ No<br>◯ Not applicable<br><br>iii) ◯ Yes ◯ No<br>◯ Not applicable<br><br>iv) ◯ Yes ◯ No<br>◯ Not applicable<br><br>v) ◯ Yes ◯ No<br>◯ Not applicable	i) ◯ Yes ◯ No<br>◯ Not applicable<br><br>ii) ◯ Yes ◯ No<br>◯ Not applicable<br><br>iii) ◯ Yes ◯ No<br>◯ Not applicable<br><br>iv) ◯ Yes ◯ No<br>◯ Not applicable<br><br>v) ◯ Yes ◯ No<br>◯ Not applicable	i) ◯ Yes ◯ No<br>◯ Not applicable<br><br>ii) ◯ Yes ◯ No<br>◯ Not applicable<br><br>iii) ◯ Yes ◯ No<br>◯ Not applicable<br><br>iv) ◯ Yes ◯ No<br>◯ Not applicable<br><br>v) ◯ Yes ◯ No<br>◯ Not applicable

**Item 18a**	**i) Did the Crisis Team ask you about any physical health problems you might have had?**				
**Items 18b, 18c**	**ii) If there were any, did they provide you with any help or offer any extra tests?**				
**SU1**	**SU2**	**SU3**	**SU4**	**SU5**	**SU6**
i) ◯ Yes ◯ No<br><br>ii) ◯ Yes ◯ No<br>◯ Left it to my GP	i) ◯ Yes ◯ No<br><br>ii) ◯ Yes ◯ No<br>◯ Left it to my GP	i) ◯ Yes ◯ No<br><br>ii) ◯ Yes ◯ No<br>◯ Left it to my GP	i) ◯ Yes ◯ No<br><br>ii) ◯ Yes ◯ No<br>◯ Left it to my GP	i) ◯ Yes ◯ No<br><br>ii) ◯ Yes ◯ No<br>◯ Left it to my GP	i) ◯ Yes ◯ No<br><br>ii) ◯ Yes ◯ No<br>◯ Left it to my GP

**Item 19**	**Did the Crisis Team help you with any practical problems you might have? (Note: 'No' should only be ticked if there were problems with any of these areas that the Crisis Team did not help with, otherwise 'Not applicable' should be used.)**<br><br>**i) Basic living needs (getting food, heating, cleaning, repairs)**<br>**ii) Benefits or debts**<br>**iii) Urgent legal or social problems (including employment, housing, etc.)**				
**SU1**	**SU2**	**SU3**	**SU4**	**SU5**	**SU6**
i) ◯ Yes ◯ No<br>◯ Not applicable<br><br>ii) ◯ Yes ◯ No<br>◯ Not applicable<br><br>iii) ◯ Yes ◯ No<br>◯ Not applicable	i) ◯ Yes ◯ No<br>◯ Not applicable<br><br>ii) ◯ Yes ◯ No<br>◯ Not applicable<br><br>iii) ◯ Yes ◯ No<br>◯ Not applicable	i) ◯ Yes ◯ No<br>◯ Not applicable<br><br>ii) ◯ Yes ◯ No<br>◯ Not applicable<br><br>iii) ◯ Yes ◯ No<br>◯ Not applicable	i) ◯ Yes ◯ No<br>◯ Not applicable<br><br>ii) ◯ Yes ◯ No<br>◯ Not applicable<br><br>iii) ◯ Yes ◯ No<br>◯ Not applicable	i) ◯ Yes ◯ No<br>◯ Not applicable<br><br>ii) ◯ Yes ◯ No<br>◯ Not applicable<br><br>iii) ◯ Yes ◯ No<br>◯ Not applicable	i) ◯ Yes ◯ No<br>◯ Not applicable<br><br>ii) ◯ Yes ◯ No<br>◯ Not applicable<br><br>iii) ◯ Yes ◯ No<br>◯ Not applicable

Item 20b	Did you feel that the treatment and support reflected your needs and what you wanted?				
**SU1**	**SU2**	**SU3**	**SU4**	**SU5**	**SU6**
i) ◯ Yes ◯ No	i) ◯ Yes ◯ No	i) ◯ Yes ◯ No	i) ◯ Yes ◯ No	i) ◯ Yes ◯ No	i) ◯ Yes ◯ No

Item 21c	i) How long did the Crisis Team normally stay when they visited (on average)?				
**Item 21d**	**ii) Did you feel that the visits were long enough for you to be able to discuss things you were concerned about?**				
**SU1**	**SU2**	**SU3**	**SU4**	**SU5**	**SU6**
i) ◯ Less than 30 minutes<br>◯ ½ to 1 hour<br>◯ More than 1 hour<br><br>ii) ◯ Yes ◯ No	i) ◯ Less than 30 minutes<br>◯ ½ to 1 hour<br>◯ More than 1 hour<br><br>ii) ◯ Yes ◯ No	i) ◯ Less than 30 minutes<br>◯ ½ to 1 hour<br>◯ More than 1 hour<br><br>ii) ◯ Yes ◯ No	i) ◯ Less than 30 minutes<br>◯ ½ to 1 hour<br>◯ More than 1 hour<br><br>ii) ◯ Yes ◯ No	i) ◯ Less than 30 minutes<br>◯ ½ to 1 hour<br>◯ More than 1 hour<br><br>ii) ◯ Yes ◯ No	i) ◯ Less than 30 minutes<br>◯ ½ to 1 hour<br>◯ More than 1 hour<br><br>ii) ◯ Yes ◯ No

**Item 22c**	**i) Have you ever been asked to feed back to the Crisis Team, for example in a survey or questionnaire, how you found them?**
**Item 22e**	**ii) Did you feel that the staff who visited you were kind, respectful, understanding?**
**Item 22e**	**iii) Did you feel listened to?**

**SU1**	**SU2**	**SU3**	**SU4**	**SU5**	**SU6**
i) ◯ Yes ◯ No<br>ii) ◯ Yes ◯ No<br>iii) ◯ Yes ◯ No	i) ◯ Yes ◯ No<br>ii) ◯ Yes ◯ No<br>iii) ◯ Yes ◯ No	i) ◯ Yes ◯ No<br>ii) ◯ Yes ◯ No<br>iii) ◯ Yes ◯ No	i) ◯ Yes ◯ No<br>ii) ◯ Yes ◯ No<br>iii) ◯ Yes ◯ No	i) ◯ Yes ◯ No<br>ii) ◯ Yes ◯ No<br>iii) ◯ Yes ◯ No	i) ◯ Yes ◯ No<br>ii) ◯ Yes ◯ No<br>iii) ◯ Yes ◯ No

**Item 23c**	**i) Were the Crisis Team flexible about where you met?**
**Item 23d**	**ii) Were they flexible about the timing of visits?**

**SU1**	**SU2**	**SU3**	**SU4**	**SU5**	**SU6**
i) ◯ Yes ◯ No<br>ii) ◯ Yes ◯ No	i) ◯ Yes ◯ No<br>ii) ◯ Yes ◯ No	i) ◯ Yes ◯ No<br>ii) ◯ Yes ◯ No	i) ◯ Yes ◯ No<br>ii) ◯ Yes ◯ No	i) ◯ Yes ◯ No<br>ii) ◯ Yes ◯ No	i) ◯ Yes ◯ No<br>ii) ◯ Yes ◯ No

	**Did the Crisis Team discuss with you or assist you with any of the following?**
**Item 24a**	**i) A personal relapse prevention plan (e.g. something that focuses mainly on identifying and monitoring early warning signs of relapse)**
**Item 24b**	**ii) A structured self-management programme (e.g. WRAP, anxiety management – resources that focus on how to stay well)**
**Item 24c**	**iii) An advance directive (e.g. information about how you want to be treated, should you become ill)**

**SU1**	**SU2**	**SU3**	**SU4**	**SU5**	**SU6**
i) ◯ Yes ◯ No<br>ii) ◯ Yes ◯ No<br>iii) ◯ Yes ◯ No	i) ◯ Yes ◯ No<br>ii) ◯ Yes ◯ No<br>iii) ◯ Yes ◯ No	i) ◯ Yes ◯ No<br>ii) ◯ Yes ◯ No<br>iii) ◯ Yes ◯ No	i) ◯ Yes ◯ No<br>ii) ◯ Yes ◯ No<br>iii) ◯ Yes ◯ No	i) ◯ Yes ◯ No<br>ii) ◯ Yes ◯ No<br>iii) ◯ Yes ◯ No	i) ◯ Yes ◯ No<br>ii) ◯ Yes ◯ No<br>iii) ◯ Yes ◯ No

Item 25c	i) Did the Crisis Team arrange a discharge meeting with you?
Items 25c, 25d	ii) If you had a discharge meeting, did anyone else attend?
Item 25e	iii) Did they give you a written discharge plan?
Item 25f	iv) Did they give you details of how you could access crisis help in the future if you needed it?

SU1	SU2	SU3	SU4	SU5	SU6
i) ◯ Yes ◯ No<br><br>ii) ◯ Family<br>◯ Friend<br>◯ Anyone from another service?<br><br>iii) ◯ Yes ◯ No<br><br>iv) ◯ Yes ◯ No	i) ◯ Yes ◯ No<br><br>ii) ◯ Family<br>◯ Friend<br>◯ Anyone from another service?<br><br>iii) ◯ Yes ◯ No<br><br>iv) ◯ Yes ◯ No	i) ◯ Yes ◯ No<br><br>ii) ◯ Family<br>◯ Friend<br>◯ Anyone from another service?<br><br>iii) ◯ Yes ◯ No<br><br>iv) ◯ Yes ◯ No	i) ◯ Yes ◯ No<br><br>ii) ◯ Family<br>◯ Friend<br>◯ Anyone from another service?<br><br>iii) ◯ Yes ◯ No<br><br>iv) ◯ Yes ◯ No	i) ◯ Yes ◯ No<br><br>ii) ◯ Family<br>◯ Friend<br>◯ Anyone from another service?<br><br>iii) ◯ Yes ◯ No<br><br>iv) ◯ Yes ◯ No	i) ◯ Yes ◯ No<br><br>ii) ◯ Family<br>◯ Friend<br>◯ Anyone from another service?<br><br>iii) ◯ Yes ◯ No<br><br>iv) ◯ Yes ◯ No

**Item 26a**	**i) How much notice did the Crisis Team give you about when you would stop seeing them?**
**Item 26b**	**ii) Did they discuss with you about how and when care from them should end?**
**Item 26c**	**iii) Did they discuss with you the idea of gradually decreasing their support?**
**Item 26d**	**iv) Were you able to contact the Crisis Team once you had been discharged if you needed to?**
**Item 26e**	**v) Did they give you any information about other local services or resources you could use?**

**SU1**	**SU2**	**SU3**	**SU4**	**SU5**	**SU6**
i) ◯ More than 48 hours<br>◯ Less than 48 hours<br>◯ No notice<br><br>ii) ◯ Yes ◯ No<br><br>iii) ◯ Yes ◯ No<br><br>iv) ◯ Yes ◯ No<br><br>v) ◯ Yes ◯ No	i) ◯ More than 48 hours<br>◯ Less than 48 hours<br>◯ No notice<br><br>ii) ◯ Yes ◯ No<br><br>iii) ◯ Yes ◯ No<br><br>iv) ◯ Yes ◯ No<br><br>v) ◯ Yes ◯ No	i) ◯ More than 48 hours<br>◯ Less than 48 hours<br>◯ No notice<br><br>ii) ◯ Yes ◯ No<br><br>iii) ◯ Yes ◯ No<br><br>iv) ◯ Yes ◯ No<br><br>v) ◯ Yes ◯ No	i) ◯ More than 48 hours<br>◯ Less than 48 hours<br>◯ No notice<br><br>ii) ◯ Yes ◯ No<br><br>iii) ◯ Yes ◯ No<br><br>iv) ◯ Yes ◯ No<br><br>v) ◯ Yes ◯ No	i) ◯ More than 48 hours<br>◯ Less than 48 hours<br>◯ No notice<br><br>ii) ◯ Yes ◯ No<br><br>iii) ◯ Yes ◯ No<br><br>iv) ◯ Yes ◯ No<br><br>v) ◯ Yes ◯ No	i) ◯ More than 48 hours<br>◯ Less than 48 hours<br>◯ No notice<br><br>ii) ◯ Yes ◯ No<br><br>iii) ◯ Yes ◯ No<br><br>iv) ◯ Yes ◯ No<br><br>v) ◯ Yes ◯ No

Item 34a	i) Did you have anyone from other mental health services involved with you while the Crisis Team were supporting you?<br>ii) If so, did you have anyone from another service attend a meeting with you and the Crisis Team together?				
**SU1**	**SU2**	**SU3**	**SU4**	**SU5**	**SU6**
i) ◯ Yes ◯ No<br>ii) ◯ Yes ◯ No	i) ◯ Yes ◯ No<br>ii) ◯ Yes ◯ No	i) ◯ Yes ◯ No<br>ii) ◯ Yes ◯ No	i) ◯ Yes ◯ No<br>ii) ◯ Yes ◯ No	i) ◯ Yes ◯ No<br>ii) ◯ Yes ◯ No	i) ◯ Yes ◯ No<br>ii) ◯ Yes ◯ No

Item 36b	i) Did the Crisis Team allocate you a named worker?				
**Item 36d**	**ii) Did everyone you met seem to know about you and your needs (or did you have to keep repeating yourself with different people)?**				
**SU1**	**SU2**	**SU3**	**SU4**	**SU5**	**SU6**
i) ◯ Yes ◯ No<br>◯ Not applicable<br>ii) ◯ Yes ◯ No	i) ◯ Yes ◯ No<br>◯ Not applicable<br>ii) ◯ Yes ◯ No	i) ◯ Yes ◯ No<br>◯ Not applicable<br>ii) ◯ Yes ◯ No	i) ◯ Yes ◯ No<br>◯ Not applicable<br>ii) ◯ Yes ◯ No	i) ◯ Yes ◯ No<br>◯ Not applicable<br>ii) ◯ Yes ◯ No	i) ◯ Yes ◯ No<br>◯ Not applicable<br>ii) ◯ Yes ◯ No

**Item 38b**	**Did the Crisis Team ever visit you more than twice a day?**					
**SU1**		**SU2**	**SU3**	**SU4**	**SU5**	**SU6**
◯ Yes ◯ No		◯ Yes ◯ No	◯ Yes ◯ No	◯ Yes ◯ No	◯ Yes ◯ No	◯ Yes ◯ No

**Item 39**	**i) Did the Crisis Team mostly see you at home?**<br>**ii) If not, was that your choice**?					
**SU1**		**SU2**	**SU3**	**SU4**	**SU5**	**SU6**
i) ◯ Yes ◯ No<br>ii) ◯ Yes ◯ No		i) ◯ Yes ◯ No<br>ii) ◯ Yes ◯ No	i) ◯ Yes ◯ No<br>ii) ◯ Yes ◯ No	i) ◯ Yes ◯ No<br>ii) ◯ Yes ◯ No	i) ◯ Yes ◯ No<br>ii) ◯ Yes ◯ No	i) ◯ Yes ◯ No<br>ii) ◯ Yes ◯ No
