# Supplementary material for: Similarities and differences between service users’ and carers’ experiences of crisis resolution teams in Norway: a survey
Source: BMC Psychiatry. 2022 Apr 14;22:266. doi: 10.1186/s12888-022-03928-w (PMC9011940; doi:10.1186/s12888-022-03928-w)
Supplement: Supplementary file 2 — Additional file 2. [file 12888_2022_3928_MOESM2_ESM.pdf]

CORE CRT Fidelity Review - Carer interview schedule

**Item 1**	**How long was it between being referred and seeing the Crisis Team to be assessed?**				
**C1**	**C2**	**C3**	**C4**	**C5**	**C6**
◯ Within 4 hours<br>◯ The same day<br>◯ Next day or later	◯ Within 4 hours<br>◯ The same day<br>◯ Next day or later	◯ Within 4 hours<br>◯ The same day<br>◯ Next day or later	◯ Within 4 hours<br>◯ The same day<br>◯ Next day or later	◯ Within 4 hours<br>◯ The same day<br>◯ Next day or later	◯ Within 4 hours<br>◯ The same day<br>◯ Next day or later

**Item 3**	**Who referred the person you care for to the Crisis Team?**				
**C1**	**C2**	**C3**	**C4**	**C5**	**C6**
◯ Staff on a Ward you were on<br>◯ Yourself<br>◯ Family or friends<br>◯ A&E<br>◯ GP<br>◯ Another service:	◯ Staff on a Ward you were on<br>◯ Yourself<br>◯ Family or friends<br>◯ A&E<br>◯ GP<br>◯ Another service:	◯ Staff on a Ward you were on<br>◯ Yourself<br>◯ Family or friends<br>◯ A&E<br>◯ GP<br>◯ Another service:	◯ Staff on a Ward you were on<br>◯ Yourself<br>◯ Family or friends<br>◯ A&E<br>◯ GP<br>◯ Another service:	◯ Staff on a Ward you were on<br>◯ Yourself<br>◯ Family or friends<br>◯ A&E<br>◯ GP<br>◯ Another service:	◯ Staff on a Ward you were on<br>◯ Yourself<br>◯ Family or friends<br>◯ A&E<br>◯ GP<br>◯ Another service:

**Item 5**	**i) Did the person you care for ever need urgent help during the night from the Crisis Team?**<br>**ii) If Yes – were they able to help on the phone?**<br>**iii) Did they ever offer to visit in person during the night?**				
**C1**	**C2**	**C3**	**C4**	**C5**	**C6**
i) ◯ Yes ◯ No<br>ii) ◯ Yes ◯ No<br>iii) ◯ Yes ◯ No	i) ◯ Yes ◯ No<br>ii) ◯ Yes ◯ No<br>iii) ◯ Yes ◯ No	i) ◯ Yes ◯ No<br>ii) ◯ Yes ◯ No<br>iii) ◯ Yes ◯ No	i) ◯ Yes ◯ No<br>ii) ◯ Yes ◯ No<br>iii) ◯ Yes ◯ No	i) ◯ Yes ◯ No<br>ii) ◯ Yes ◯ No<br>iii) ◯ Yes ◯ No	i) ◯ Yes ◯ No<br>ii) ◯ Yes ◯ No<br>iii) ◯ Yes ◯ No

**Item 2b**	**i) Were you or the person you care for given a direct phone number to contact the Crisis Team?**				
**Items 9a, 9c, 9d**	**ii) Did you or the person you care for use it?**<br>**iii) If Yes - did the Crisis Team answer the phone in person?**<br>**iv) If Yes – how quickly were you or the person you care for able to talk to clinical staff?**				
**C1**	**C2**	**C3**	**C4**	**C5**	**C6**
i) ◯ Yes ◯ No<br>ii) ◯ Yes ◯ No<br>iii) ◯ Yes ◯ No<br>iv) ◯ Straightaway<br>◯ within 20 minutes<br>◯ within 1 hour<br>◯ more than 1 hour	i) ◯ Yes ◯ No<br>ii) ◯ Yes ◯ No<br>iii) ◯ Yes ◯ No<br>iv) ◯ Straightaway<br>◯ within 20 minutes<br>◯ within 1 hour<br>◯ more than 1 hour	i) ◯ Yes ◯ No<br>ii) ◯ Yes ◯ No<br>iii) ◯ Yes ◯ No<br>iv) ◯ Straightaway<br>◯ within 20 minutes<br>◯ within 1 hour<br>◯ more than 1 hour	i) ◯ Yes ◯ No<br>ii) ◯ Yes ◯ No<br>iii) ◯ Yes ◯ No<br>iv) ◯ Straightaway<br>◯ within 20 minutes<br>◯ within 1 hour<br>◯ more than 1 hour	i) ◯ Yes ◯ No<br>ii) ◯ Yes ◯ No<br>iii) ◯ Yes ◯ No<br>iv) ◯ Straightaway<br>◯ within 20 minutes<br>◯ within 1 hour<br>◯ more than 1 hour	i) ◯ Yes ◯ No<br>ii) ◯ Yes ◯ No<br>iii) ◯ Yes ◯ No<br>iv) ◯ Straightaway<br>◯ within 20 minutes<br>◯ within 1 hour<br>◯ more than 1 hour

Item 9e	i) **Did you or the person you care for ever ask the Crisis Team to come back a second time on a day when they had already visited you?**<br>**ii) If Yes – were they able to do this?**				
**C1**	**C2**	**C3**	**C4**	**C5**	**C6**
i) ◯ Yes ◯ No<br>ii) ◯ Yes ◯ No	i) ◯ Yes ◯ No<br>ii) ◯ Yes ◯ No	i) ◯ Yes ◯ No<br>ii) ◯ Yes ◯ No	i) ◯ Yes ◯ No<br>ii) ◯ Yes ◯ No	i) ◯ Yes ◯ No<br>ii) ◯ Yes ◯ No	i) ◯ Yes ◯ No<br>ii) ◯ Yes ◯ No

**Item 12a**	**i) When the Crisis Team saw the person you care for initially did they provide information about their role and how they would help?**
**Item 12a**	**ii) Was any of this information in writing?**
**Item 12b**	**iii) Did they give you or the person you care for a written treatment plan?**
**Items 12c, 12d**	**iv) Generally, did they let you or the person you care for know in advance when they would be visiting?**
**Item 12e**	**v) Did they turn up reasonably on time (within an hour)?**
**Item 12f**	**vi) If they were going to be late, did they contact you or the person you care for to let you know?**

	**C1**	**C2**	**C3**	**C4**	**C5**	**C6**
i)	◯ Yes ◯ No	◯ Yes ◯ No	◯ Yes ◯ No	◯ Yes ◯ No	◯ Yes ◯ No	◯ Yes ◯ No
ii)	◯ Yes ◯ No	◯ Yes ◯ No	◯ Yes ◯ No	◯ Yes ◯ No	◯ Yes ◯ No	◯ Yes ◯ No
iii)	◯ Yes, within 4 days<br>◯ Yes, but later<br>◯ No	◯ Yes, within 4 days<br>◯ Yes, but later<br>◯ No	◯ Yes, within 4 days<br>◯ Yes, but later<br>◯ No	◯ Yes, within 4 days<br>◯ Yes, but later<br>◯ No	◯ Yes, within 4 days<br>◯ Yes, but later<br>◯ No	◯ Yes, within 4 days<br>◯ Yes, but later<br>◯ No
iv)	◯ At least a day before<br>◯ On the same day<br>◯ Turned up by surprise	◯ At least a day before<br>◯ On the same day<br>◯ Turned up by surprise	◯ At least a day before<br>◯ On the same day<br>◯ Turned up by surprise	◯ At least a day before<br>◯ On the same day<br>◯ Turned up by surprise	◯ At least a day before<br>◯ On the same day<br>◯ Turned up by surprise	◯ At least a day before<br>◯ On the same day<br>◯ Turned up by surprise
v)	◯ Yes ◯ No	◯ Yes ◯ No	◯ Yes ◯ No	◯ Yes ◯ No	◯ Yes ◯ No	◯ Yes ◯ No
vi)	◯ Always<br>◯ Usually<br>◯ Never	◯ Always<br>◯ Usually<br>◯ Never	◯ Always<br>◯ Usually<br>◯ Never	◯ Always<br>◯ Usually<br>◯ Never	◯ Always<br>◯ Usually<br>◯ Never	◯ Always<br>◯ Usually<br>◯ Never

Item 13	i) Was the person you care for willing for you to be involved with their care?
Item 13e	ii) If Yes – overall did the Crisis Team involve you in supporting him/her?
Item 13c	iii) Were you (or anyone else supporting the person you care for) invited to a care planning or review meeting with the Crisis Team?

C1	C2	C3	C4	C5	C6
i) ◯ Yes ◯ No	i) ◯ Yes ◯ No	i) ◯ Yes ◯ No	i) ◯ Yes ◯ No	i) ◯ Yes ◯ No	i) ◯ Yes ◯ No
ii) ◯ Yes ◯ No	ii) ◯ Yes ◯ No	ii) ◯ Yes ◯ No	ii) ◯ Yes ◯ No	ii) ◯ Yes ◯ No	ii) ◯ Yes ◯ No
iii) ◯ Yes ◯ No	iii) ◯ Yes ◯ No	iii) ◯ Yes ◯ No	iii) ◯ Yes ◯ No	iii) ◯ Yes ◯ No	iii) ◯ Yes ◯ No

Item 14a	i) Did the Crisis Team staff offer to meet with you on your own, separately from the person you care for?
Item 14b	ii) Did they provide you with information about local resources for carers such as carers' groups or welfare advice)?
Item 14d	iii) Did they provide you with your own support plan?

C1	C2	C3	C4	C5	C6
i) ◯ Yes ◯ No	i) ◯ Yes ◯ No	i) ◯ Yes ◯ No	i) ◯ Yes ◯ No	i) ◯ Yes ◯ No	i) ◯ Yes ◯ No
ii) ◯ Yes ◯ No	ii) ◯ Yes ◯ No	ii) ◯ Yes ◯ No	ii) ◯ Yes ◯ No	ii) ◯ Yes ◯ No	ii) ◯ Yes ◯ No
iii) ◯ Yes ◯ No	iii) ◯ Yes ◯ No	iii) ◯ Yes ◯ No	iii) ◯ Yes ◯ No	iii) ◯ Yes ◯ No	iii) ◯ Yes ◯ No

Items 15b, 15c	i) Did the Crisis Team bring medication for the person you care for when needed?				
**Item 15c**	**ii) How often did they bring medication?**				
**C1**	**C2**	**C3**	**C4**	**C5**	**C6**
i) ◯ Yes ◯ No<br>◯ Not applicable<br><br>ii) ◯ Daily<br>◯ More than once daily<br>◯ Occasionally<br><br>iii) ◯ Yes ◯ No	i) ◯ Yes ◯ No<br>◯ Not applicable<br><br>ii) ◯ Daily<br>◯ More than once daily<br>◯ Occasionally<br><br>iii) ◯ Yes ◯ No	i) ◯ Yes ◯ No<br>◯ Not applicable<br><br>ii) ◯ Daily<br>◯ More than once daily<br>◯ Occasionally<br><br>iii) ◯ Yes ◯ No	i) ◯ Yes ◯ No<br>◯ Not applicable<br><br>ii) ◯ Daily<br>◯ More than once daily<br>◯ Occasionally<br><br>iii) ◯ Yes ◯ No	i) ◯ Yes ◯ No<br>◯ Not applicable<br><br>ii) ◯ Daily<br>◯ More than once daily<br>◯ Occasionally<br><br>iii) ◯ Yes ◯ No	i) ◯ Yes ◯ No<br>◯ Not applicable<br><br>ii) ◯ Daily<br>◯ More than once daily<br>◯ Occasionally<br><br>iii) ◯ Yes ◯ No

**Item 16d**	**i) Did they give you or the person you care for a list of what medication they should be taking and when?**
**Item 16e**	**ii) Did the Crisis Team help you and the person you care for to understand any medication prescribed – such as what it was for or any possible side-effects?**
**Item 16e**	**iii) Did they give any of this information in writing?**
**Item 16f**	**iv) Did the person you care for have any problems with taking medication regularly and on time?**
**Item 16f**	**v) If Yes - did the Crisis Team do anything to help with this?**

**C1**	**C2**	**C3**	**C4**	**C5**	**C6**
i) ◯ Yes ◯ No<br>◯ Not applicable<br><br>ii) ◯ Yes ◯ No<br>◯ Not applicable<br><br>iii) ◯ Yes ◯ No<br>◯ Not applicable<br><br>iv) ◯ Yes ◯ No<br>◯ Not applicable<br><br>v) ◯ Yes ◯ No<br>◯ Not applicable	i) ◯ Yes ◯ No<br>◯ Not applicable<br><br>ii) ◯ Yes ◯ No<br>◯ Not applicable<br><br>iii) ◯ Yes ◯ No<br>◯ Not applicable<br><br>iv) ◯ Yes ◯ No<br>◯ Not applicable<br><br>v) ◯ Yes ◯ No<br>◯ Not applicable	i) ◯ Yes ◯ No<br>◯ Not applicable<br><br>ii) ◯ Yes ◯ No<br>◯ Not applicable<br><br>iii) ◯ Yes ◯ No<br>◯ Not applicable<br><br>iv) ◯ Yes ◯ No<br>◯ Not applicable<br><br>v) ◯ Yes ◯ No<br>◯ Not applicable	i) ◯ Yes ◯ No<br>◯ Not applicable<br><br>ii) ◯ Yes ◯ No<br>◯ Not applicable<br><br>iii) ◯ Yes ◯ No<br>◯ Not applicable<br><br>iv) ◯ Yes ◯ No<br>◯ Not applicable<br><br>v) ◯ Yes ◯ No<br>◯ Not applicable	i) ◯ Yes ◯ No<br>◯ Not applicable<br><br>ii) ◯ Yes ◯ No<br>◯ Not applicable<br><br>iii) ◯ Yes ◯ No<br>◯ Not applicable<br><br>iv) ◯ Yes ◯ No<br>◯ Not applicable<br><br>v) ◯ Yes ◯ No<br>◯ Not applicable	i) ◯ Yes ◯ No<br>◯ Not applicable<br><br>ii) ◯ Yes ◯ No<br>◯ Not applicable<br><br>iii) ◯ Yes ◯ No<br>◯ Not applicable<br><br>iv) ◯ Yes ◯ No<br>◯ Not applicable<br><br>v) ◯ Yes ◯ No<br>◯ Not applicable

Item 18a	i) Did the Crisis Team ask about any physical health problems the person you care for might have had?
Item 18b, 18c	ii) If there were any, did they provide any help with this or offer any extra tests?

C1	C2	C3	C4	C5	C6
i) ◯ Yes ◯ No<br><br>ii) ◯ Yes ◯ No<br>◯ Left it to my GP	i) ◯ Yes ◯ No<br><br>ii) ◯ Yes ◯ No<br>◯ Left it to my GP	i) ◯ Yes ◯ No<br><br>ii) ◯ Yes ◯ No<br>◯ Left it to my GP	i) ◯ Yes ◯ No<br><br>ii) ◯ Yes ◯ No<br>◯ Left it to my GP	i) ◯ Yes ◯ No<br><br>ii) ◯ Yes ◯ No<br>◯ Left it to my GP	i) ◯ Yes ◯ No<br><br>ii) ◯ Yes ◯ No<br>◯ Left it to my GP

Item 19	Did the Crisis Team help you and the person you care for with any practical problems? (Note: 'No' should only be ticked if there were problems with any of these areas that the Crisis Team did not help with, otherwise 'Not applicable' should be used.)<br><br>i) Basic living needs (getting food, heating, cleaning, repairs)<br>ii) Benefits or debts<br>iii) Urgent legal or social problems (including employment, housing, etc.)

C1	C2	C3	C4	C5	C6
i) ◯ Yes ◯ No<br>◯ Not applicable<br><br>ii) ◯ Yes ◯ No<br>◯ Not applicable<br><br>iii) ◯ Yes ◯ No<br>◯ Not applicable	i) ◯ Yes ◯ No<br>◯ Not applicable<br><br>ii) ◯ Yes ◯ No<br>◯ Not applicable<br><br>iii) ◯ Yes ◯ No<br>◯ Not applicable	i) ◯ Yes ◯ No<br>◯ Not applicable<br><br>ii) ◯ Yes ◯ No<br>◯ Not applicable<br><br>iii) ◯ Yes ◯ No<br>◯ Not applicable	i) ◯ Yes ◯ No<br>◯ Not applicable<br><br>ii) ◯ Yes ◯ No<br>◯ Not applicable<br><br>iii) ◯ Yes ◯ No<br>◯ Not applicable	i) ◯ Yes ◯ No<br>◯ Not applicable<br><br>ii) ◯ Yes ◯ No<br>◯ Not applicable<br><br>iii) ◯ Yes ◯ No<br>◯ Not applicable	i) ◯ Yes ◯ No<br>◯ Not applicable<br><br>ii) ◯ Yes ◯ No<br>◯ Not applicable<br><br>iii) ◯ Yes ◯ No<br>◯ Not applicable

Item 20b	Did you feel that the treatment and support reflected the needs of the person you care for and what they wanted?				
**C1**	**C2**	**C3**	**C4**	**C5**	**C6**
i) ◯ Yes ◯ No	i) ◯ Yes ◯ No	i) ◯ Yes ◯ No	i) ◯ Yes ◯ No	i) ◯ Yes ◯ No	i) ◯ Yes ◯ No

Item 21c	i) How long did the Crisis Team normally stay when they visited (on average)?				
**Item 21d**	**ii) Did you feel that the visits were long enough for you to be able to discuss things you or the person you care for were concerned about?**				
**C1**	**C2**	**C3**	**C4**	**C5**	**C6**
i) ◯ Less than 30 minutes<br>◯ ½ to 1 hour<br>◯ More than 1 hour<br><br>ii) ◯ Yes ◯ No	i) ◯ Less than 30 minutes<br>◯ ½ to 1 hour<br>◯ More than 1 hour<br><br>ii) ◯ Yes ◯ No	i) ◯ Less than 30 minutes<br>◯ ½ to 1 hour<br>◯ More than 1 hour<br><br>ii) ◯ Yes ◯ No	i) ◯ Less than 30 minutes<br>◯ ½ to 1 hour<br>◯ More than 1 hour<br><br>ii) ◯ Yes ◯ No	i) ◯ Less than 30 minutes<br>◯ ½ to 1 hour<br>◯ More than 1 hour<br><br>ii) ◯ Yes ◯ No	i) ◯ Less than 30 minutes<br>◯ ½ to 1 hour<br>◯ More than 1 hour<br><br>ii) ◯ Yes ◯ No

**Item 22c**	**i) Have you ever been asked to feed back to the Crisis Team, for example in a survey or questionnaire, how you found them?**
**Item 22e**	**ii) Did you feel that the staff who visited were kind, respectful, understanding?**
**Item 22e**	**iii) Did you feel listened to?**

**C1**	**C2**	**C3**	**C4**	**C5**	**C6**
i) ◯ Yes ◯ No	i) ◯ Yes ◯ No	i) ◯ Yes ◯ No	i) ◯ Yes ◯ No	i) ◯ Yes ◯ No	i) ◯ Yes ◯ No
ii) ◯ Yes ◯ No	ii) ◯ Yes ◯ No	ii) ◯ Yes ◯ No	ii) ◯ Yes ◯ No	ii) ◯ Yes ◯ No	ii) ◯ Yes ◯ No
iii) ◯ Yes ◯ No	iii) ◯ Yes ◯ No	iii) ◯ Yes ◯ No	iii) ◯ Yes ◯ No	iii) ◯ Yes ◯ No	iii) ◯ Yes ◯ No

**Item 23c**	**i) Were the Crisis Team flexible about where you met?**
**Item 23d**	**ii) Were they flexible about the timing of visits?**

**C1**	**C2**	**C3**	**C4**	**C5**	**C6**
i) ◯ Yes ◯ No	i) ◯ Yes ◯ No	i) ◯ Yes ◯ No	i) ◯ Yes ◯ No	i) ◯ Yes ◯ No	i) ◯ Yes ◯ No
ii) ◯ Yes ◯ No	ii) ◯ Yes ◯ No	ii) ◯ Yes ◯ No	ii) ◯ Yes ◯ No	ii) ◯ Yes ◯ No	ii) ◯ Yes ◯ No

	**Did the Crisis Team discuss with the person you care for or assist with any of the following?**
**Item 24a**	**i) A personal relapse prevention plan (e.g. something that focuses mainly on identifying and monitoring early warning signs of relapse)**
**Item 24b**	**ii) A structured self-management programme (e.g. WRAP, anxiety management – resources that focus on how to stay well)**
**Item 24c**	**iii) An advance directive (e.g. information about the person you care for wants to be treated, should they become ill)**

**C1**	**C2**	**C3**	**C4**	**C5**	**C6**
i) ◯ Yes ◯ No	i) ◯ Yes ◯ No	i) ◯ Yes ◯ No	i) ◯ Yes ◯ No	i) ◯ Yes ◯ No	i) ◯ Yes ◯ No
ii) ◯ Yes ◯ No	ii) ◯ Yes ◯ No	ii) ◯ Yes ◯ No	ii) ◯ Yes ◯ No	ii) ◯ Yes ◯ No	ii) ◯ Yes ◯ No
iii) ◯ Yes ◯ No	iii) ◯ Yes ◯ No	iii) ◯ Yes ◯ No	iii) ◯ Yes ◯ No	iii) ◯ Yes ◯ No	iii) ◯ Yes ◯ No

**Item 25c**	**i) Did the Crisis Team arrange a discharge meeting with you and the person you care for?**
**Items 25c, 25d**	**ii) If there was a discharge meeting, did anyone else attend?**
**Item 25e**	**iii) Did they give you a written discharge plan?**
**Item 25f**	**iv) Did they give you details of how you could access crisis help in the future if you needed it?**

**C1**	**C2**	**C3**	**C4**	**C5**	**C6**
i) ◯ Yes ◯ No<br><br>ii) ◯ Family<br>◯ Friend<br>◯ Anyone from another service?<br><br>iii) ◯ Yes ◯ No<br><br>iv) ◯ Yes ◯ No	i) ◯ Yes ◯ No<br><br>ii) ◯ Family<br>◯ Friend<br>◯ Anyone from another service?<br><br>iii) ◯ Yes ◯ No<br><br>iv) ◯ Yes ◯ No	i) ◯ Yes ◯ No<br><br>ii) ◯ Family<br>◯ Friend<br>◯ Anyone from another service?<br><br>iii) ◯ Yes ◯ No<br><br>iv) ◯ Yes ◯ No	i) ◯ Yes ◯ No<br><br>ii) ◯ Family<br>◯ Friend<br>◯ Anyone from another service?<br><br>iii) ◯ Yes ◯ No<br><br>iv) ◯ Yes ◯ No	i) ◯ Yes ◯ No<br><br>ii) ◯ Family<br>◯ Friend<br>◯ Anyone from another service?<br><br>iii) ◯ Yes ◯ No<br><br>iv) ◯ Yes ◯ No	i) ◯ Yes ◯ No<br><br>ii) ◯ Family<br>◯ Friend<br>◯ Anyone from another service?<br><br>iii) ◯ Yes ◯ No<br><br>iv) ◯ Yes ◯ No

**Item 26a**	**i) How much notice did the Crisis Team give you or the person you care for about when care from them would end?**
**Item 26b**	**ii) Did the they discuss with you or the person you care for about how and when care from them should end?**
**Item 26c**	**iii) Did they discuss with the idea of gradually decreasing their support?**
**Item 26d**	**iv) Were you or the person you care for able to contact the Crisis Team once you had been discharged if you needed to?**
**Item 26e**	**v) Did they give you any information about other local services or resources?**

**C1**	**C2**	**C3**	**C4**	**C5**	**C6**
i) ◯ More than 48 hours<br>◯ Less than 48 hours<br>◯ No notice<br><br>ii) ◯ Yes ◯ No<br><br>iii) ◯ Yes ◯ No<br><br>iv) ◯ Yes ◯ No<br><br>v) ◯ Yes ◯ No	i) ◯ More than 48 hours<br>◯ Less than 48 hours<br>◯ No notice<br><br>ii) ◯ Yes ◯ No<br><br>iii) ◯ Yes ◯ No<br><br>iv) ◯ Yes ◯ No<br><br>v) ◯ Yes ◯ No	i) ◯ More than 48 hours<br>◯ Less than 48 hours<br>◯ No notice<br><br>ii) ◯ Yes ◯ No<br><br>iii) ◯ Yes ◯ No<br><br>iv) ◯ Yes ◯ No<br><br>v) ◯ Yes ◯ No	i) ◯ More than 48 hours<br>◯ Less than 48 hours<br>◯ No notice<br><br>ii) ◯ Yes ◯ No<br><br>iii) ◯ Yes ◯ No<br><br>iv) ◯ Yes ◯ No<br><br>v) ◯ Yes ◯ No	i) ◯ More than 48 hours<br>◯ Less than 48 hours<br>◯ No notice<br><br>ii) ◯ Yes ◯ No<br><br>iii) ◯ Yes ◯ No<br><br>iv) ◯ Yes ◯ No<br><br>v) ◯ Yes ◯ No	i) ◯ More than 48 hours<br>◯ Less than 48 hours<br>◯ No notice<br><br>ii) ◯ Yes ◯ No<br><br>iii) ◯ Yes ◯ No<br><br>iv) ◯ Yes ◯ No<br><br>v) ◯ Yes ◯ No

Item 34a	i) Was there anyone from other mental health services involved while the Crisis Team were supporting the person you care for?<br>ii) If so, did anyone from another service attend a meeting with you or the person you care for and the Crisis Team together?				
**C1**	**C2**	**C3**	**C4**	**C5**	**C6**
i) ◯ Yes ◯ No<br><br>ii) ◯ Yes ◯ No	i) ◯ Yes ◯ No<br><br>ii) ◯ Yes ◯ No	i) ◯ Yes ◯ No<br><br>ii) ◯ Yes ◯ No	i) ◯ Yes ◯ No<br><br>ii) ◯ Yes ◯ No	i) ◯ Yes ◯ No<br><br>ii) ◯ Yes ◯ No	i) ◯ Yes ◯ No<br><br>ii) ◯ Yes ◯ No

Item 36b	i) Did the Crisis Team allocate a named worker to the person you care for?				
**Item 36d**	**ii) Did everyone you met seem to know about the person you care for and their needs (or did either of you have to keep repeating yourself with different people)?**				
**C1**	**C2**	**C3**	**C4**	**C5**	**C6**
i) ◯ Yes ◯ No<br>◯ Not applicable<br><br>ii) ◯ Yes ◯ No	i) ◯ Yes ◯ No<br>◯ Not applicable<br><br>ii) ◯ Yes ◯ No	i) ◯ Yes ◯ No<br>◯ Not applicable<br><br>ii) ◯ Yes ◯ No	i) ◯ Yes ◯ No<br>◯ Not applicable<br><br>ii) ◯ Yes ◯ No	i) ◯ Yes ◯ No<br>◯ Not applicable<br><br>ii) ◯ Yes ◯ No	i) ◯ Yes ◯ No<br>◯ Not applicable<br><br>ii) ◯ Yes ◯ No

Item 38b	Did the Crisis Team ever visit the person you care for more than twice a day?				
**C1**	**C2**	**C3**	**C4**	**C5**	**C6**
◯ Yes ◯ No	◯ Yes ◯ No	◯ Yes ◯ No	◯ Yes ◯ No	◯ Yes ◯ No	◯ Yes ◯ No

Item 39	i) Did the Crisis Team mostly see the person you care for at home?<br>ii) If not, was that the choice of the person you care for?				
**C1**	**C2**	**C3**	**C4**	**C5**	**C6**
i) ◯ Yes ◯ No<br>ii) ◯ Yes ◯ No	i) ◯ Yes ◯ No<br>ii) ◯ Yes ◯ No	i) ◯ Yes ◯ No<br>ii) ◯ Yes ◯ No	i) ◯ Yes ◯ No<br>ii) ◯ Yes ◯ No	i) ◯ Yes ◯ No<br>ii) ◯ Yes ◯ No	i) ◯ Yes ◯ No<br>ii) ◯ Yes ◯ No
